# A Case Report of Acute Airway Compromise due to Subcutaneous Emphysema

**DOI:** 10.1155/2018/3103061

**Published:** 2018-11-25

**Authors:** David Olmstead, Gary Gelfand, Ian Anderson, John B. Kortbeek

**Affiliations:** ^1^Cumming School of Medicine, University of Calgary, Department of Surgery, South Health Campus, 4448 Front St SE, Calgary, AB T3M1M4, Canada; ^2^Cumming School of Medicine, University of Calgary, Alberta Health Services, G33-1403 29 ST NW, Calgary, AB T2N 2T9, Canada; ^3^Cumming School of Medicine, University of Calgary, Department of Surgery, Foothills Medical Centre, Alberta Health Services, 1403 29th ST NW, Calgary, AB T2N 2T9, Canada; ^4^Cumming School of Medicine, University of Calgary, Alberta Health Services, Department of Surgery, South Health Campus, 4448 Front St SE, Calgary, AB T3M1M4, Canada

## Abstract

In the acute management of a trauma patient, airway patency is of utmost importance. The present case describes a male patient who presented with delayed severe upper airway obstruction secondary to massive subcutaneous emphysema following blunt traumatic injury two days previously. Airway compromise is a rarely described but serious complication of subcutaneous emphysema. Current management of subcutaneous emphysema and its association with pneumothorax is summarized. Early decompression of underlying pneumothoraces in patients with significant subcutaneous emphysema should be performed to avoid this rare complication.

## 1. Introduction

Subcutaneous emphysema is defined as the presence of free air in the subcutaneous tissues. Numerous causes exist for this phenomenon, including blunt and penetrating trauma, soft tissue infection, and surgical instrumentation [1]. While not necessarily hazardous, subcutaneous emphysema is known to be associated with underlying pneumothorax and, rarely, airway injury [2, 3]. One recent study involving 405 trauma patients found that, among the 24 presenting with subcutaneous emphysema on CXR, every single one had an underlying pneumothorax [4]. Airway obstruction is even more rarely described but can be life-threatening. Tobacco use, underlying respiratory disease, and recent respiratory infection have been described as potential predisposing factors for the development of mediastinal subcutaneous emphysema [1].

The present case describes a case of severe subcutaneous emphysema in a blunt trauma patient which progressed resulting in airway compromise and the need for emergent placement of an advanced surgical airway. Earlier placement of a thoracostomy tube likely would have prevented this complication.

## 2. Case Presentation

**Figure 1:**
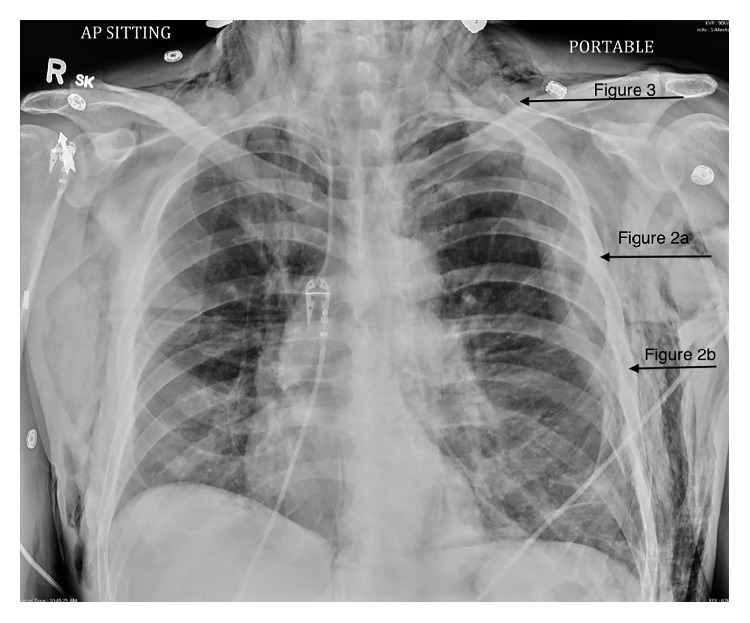
Anteroposterior chest radiograph demonstrating subcutaneous emphysema. Anatomic locations of Figures 2(a), 2(b), and 3 are indicated.

A 35-year-old male presented to a regional urban hospital two days following an assault-related blunt traumatic injury. The evening before arrival at the emergency department, he noticed swelling around his chest and neck. It was worse the next morning, precipitating his presentation to hospital. On initial assessment, the patient had a Glasgow Coma Scale of 15, and vital signs were BP 125/66, HR 92, and SpO_2_ 95% on oxygen at 5 litres per minute via nasal cannulas. At the time of presentation, the patient displayed moderate subcutaneous emphysema on physical examination and subcutaneous emphysema on chest X-ray (Figure 1).

**Figure 2:**
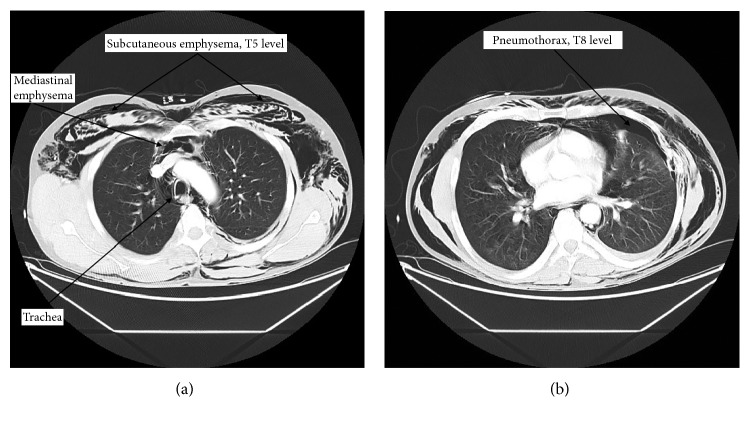
(a) Axial CT image at the T5 level demonstrating extensive subcutaneous and mediastinal emphysema. (b) Axial CT image at the T8 level demonstrating a pneumothorax on the left side. Extensive bilateral subcutaneous air is also visible.

**Figure 3:**
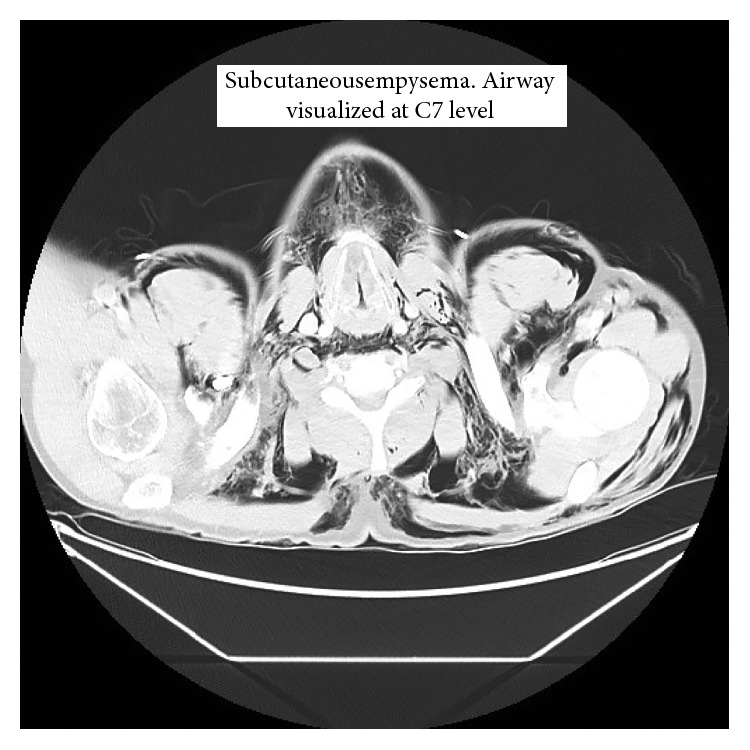
Axial CT image at the level of the thyroid cartilage demonstrating air infiltration around the larynx. There is notable swelling of the soft tissue of the larynx.

Computed tomography of the chest, abdomen, and pelvis revealed a left-sided pneumothorax and subcutaneous emphysema (Figures 2(a) and 2(b)). Significant laryngeal swelling was also noted (Figure 3). The patient was found to have multiple rib fractures, a lacerated scalp, and a Grade 1 liver laceration. A chest tube was not inserted at this time, after consultation with a thoracic surgeon at the nearby Level 1 trauma hospital. Upon reviewing the CT, it was suggested that the relatively small amount of pneumothorax for the degree of subcutaneous emphysema indicated potential pleural adhesions. The view of the thoracic surgery service and trauma was that an incorrectly placed chest tube at the regional centre may have risked entering the lung parenchyma. The patient was transferred to a Level 1 trauma centre 4 and 1/2 hours after presentation arriving 30 min later.

The extent of the subcutaneous emphysema was such that the patient could not be placed in a cervical spine collar for transport to the referral facility. His cervical spine was instead immobilized with towel rolls. Vital signs remained stable in transit, and the patient arrived at the trauma centre awake, alert, and breathing spontaneously on supplemental oxygen. The patient was assessed by the trauma service and thoracic surgery.

Over the next two hours, the patient's condition deteriorated. While the patient had been ordered to get admitted to the trauma nursing unit, the emergency room physician wisely held the patient in the high observation area of the emergency department. Seven hours after initial presentation to the regional hospital and two hours after arrival at the trauma centre, the patient demonstrated altered phonation in addition to yet greater swelling around the neck. In order to obtain a definitive airway in a controlled environment, the patient was taken to the operating room for intubation with surgical standby.

In the operating room, the patient's oxygen requirements increased, with desaturation on 10 litres per minute, now via facemask. The patient was also becoming increasingly agitated. An attempt was made at awake fiber-optic intubation, but the posterior oropharyngeal anatomy, glottis, and larynx could not be visualized. Given the increasing oxygen demands and the challenging airway, after considering all options, an awake tracheostomy was performed with a Shiley XLT extended-length tracheostomy appliance. A left thoracostomy tube was then placed. Bronchoscopy in the OR did not reveal proximal tracheobronchial injury.

The patient was transferred to the intensive care unit where he remained for 21 days. He had complications of ventilator-associated pneumonia and delirium due to substance withdrawal. A repeat bronchoscopy on day 18 was normal, and he was successfully weaned from the ventilator that day.

**Figure 4:**
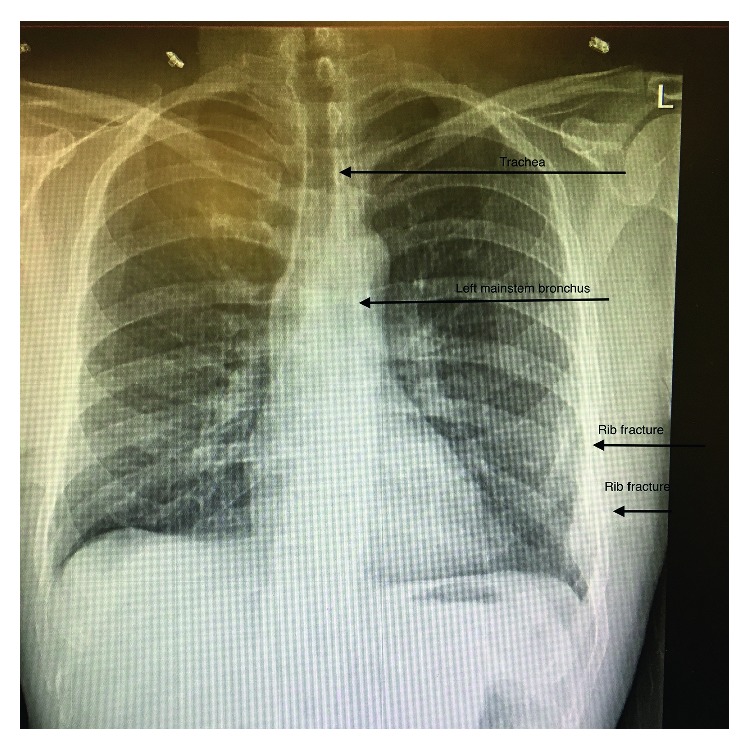
Chest radiograph from day 28 demonstrating the normal airway anatomy as well as the two left rib fractures.

Subcutaneous decompression was achieved with continued suction via the thoracostomy tube inserted in the operating room at the time of the tracheostomy. Considerable subcutaneous air was also seen escaping from the tracheostomy incision. The subcutaneous emphysema had resolved by day 14. He was transferred to the trauma ward on day 21 and decannulated on day 22. A normal CXR was performed on day 23 (Figure 4), and he was discharged on day 28.

## 3. Discussion

Subcutaneous emphysema is a common phenomenon, having been first described by Louise Bourgeois, midwife to the queen of France, in 1617. The condition was later characterized by Laennec in 1819 [5]. It has been described following injury, surgery, mechanical ventilation, and infection. There have also been case reports of patients presenting with spontaneous new onset subcutaneous emphysema for whom precipitating events cannot be identified [6].

Airway compromise resulting from free air in the subcutaneous tissues is rarely described without associated tracheal injury [7] and has the potential to be acutely life-threatening. Patients having sustained traumatic injuries to the chest wall are at risk of developing respiratory failure due to chest wall injury, pulmonary contusion or hemorrhage, or underlying pneumothorax. Airway restriction relating to the presence of their subcutaneous emphysema is rarely described as the subcutaneous air is usually easily accommodated by the distensile subcutaneous tissues. Subcutaneous emphysema's primary significance is a marker for occult pneumothorax and associated chest injuries. Prominent subcutaneous emphysema should raise suspicion of underlying tracheobronchial injury. Rarely, as in this case, the severity of subcutaneous emphysema may cause direct constriction of the proximal airway and airway obstruction. This patient also experienced hypoxemia which was almost certainly due to progressive respiratory failure associated with the underlying pneumothorax. Earlier placement of a thoracostomy tube would likely have mitigated the impending airway obstruction, the hypoxemic respiratory failure, and the progressive subcutaneous emphysema.

Fourteen case reports of airway obstruction secondary to subcutaneous emphysema have been identified in the English literature including spontaneous, surgical, and trauma cases [8–20]. Only one of these was in a trauma patient [11]. Substantial amounts of air cause epiglottic and paratracheal swelling, completely obliterating the anatomy and normal landmarks for intubation as in this case. The need to obtain cervical spinal immobilization in trauma patients may also challenge airway management. The presence of a chest tube may be an independent risk factor for the development or progression of subcutaneous emphysema if the tube is malpositioned [21].

A number of strategies have been identified in the management of subcutaneous emphysema. In the majority of cases, conservative management along with high-quality supportive care is adequate to maintain patient safety while awaiting spontaneous resolution [22–26]. Early recognition and placement of a thoracostomy tube is essential when moderate to large subcutaneous emphysema is present as the underlying pneumothorax and associated subcutaneous emphysema will progress. In cases where patient transport is required, particularly long distance and air transport, tube thoracostomy should be performed in all cases of subcutaneous emphysema to prevent the catastrophic complication of respiratory failure or tension pneumothorax in a ground or air ambulance. During transport, the opportunity for intervention is limited and the skills, equipment, and space for tube thoracostomy placement do not exist. Advanced airway management in an ambulance is also extremely challenging and may be impossible.

When compression of vital structures is noted in the neck, pleural drains and infraclavicular “gill” incisions have been reported in case studies to evacuate the subcutaneous gas [27, 28]. There have also been case reports of management with a subcutaneous negative-pressure drain [29–31]. In patients with drains already in situ, both increased suction in the existing drain as well as the insertion of a new drain have been used. To date, there is insufficient evidence to determine the relative effectiveness of subcutaneous drains, in situ chest drains, or infraclavicular incisions [32, 33].

The preferred approach in our institution and most trauma centres is early placement of a tube thoracostomy, ideally by, or supervised by, an experienced practitioner. This serves to decompress both the underlying pneumothorax, reexpand the lung, and decompress the subcutaneous emphysema. A tube thoracostomy and expectant management is usually sufficient. Subcutaneous drains do not treat the underlying pneumothorax or control the alveolar leak. Infraclavicular incisions are invasive, require wound care, cause scarring, and do not treat the underlying problem. We have not performed them.

This case highlights the importance of the initial and continuous assessment of the trauma patient. In a patient with massive subcutaneous emphysema, a previously patent airway can, rarely, become obstructed. Early recognition and placement of a thoracostomy tube, ideally performed at the hospital where the patient first presented, almost certainly would have avoided the airway obstruction. Fortunately, this patient did not deteriorate during transport. Chest trauma patients require close observation and adequate analgesia. Subcutaneous emphysema is always associated with underlying pneumothorax. Practitioners should be vigilant in identifying it and treating the pneumothorax in moderate to severe cases and those requiring transport.
